# Corneal-Based Surgical Presbyopic Therapies and Their Application in Pseudophakic Patients

**DOI:** 10.1155/2016/5263870

**Published:** 2016-03-09

**Authors:** Grace L. Paley, Roy S. Chuck, Linda M. Tsai

**Affiliations:** ^1^Department of Ophthalmology and Visual Sciences, Washington University School of Medicine, St. Louis, MO 63110, USA; ^2^Department of Ophthalmology and Visual Sciences, Albert Einstein College of Medicine and Montefiore Medical Center, Bronx, NY 10467, USA

## Abstract

*Purpose*. The purpose of this review is to provide a summary of laser refractive surgery and corneal inlay approaches to treat presbyopia in patients after cataract surgery.* Summary*. The presbyopic population is growing rapidly along with increasing demands for spectacle independence. This review will focus on the corneal-based surgical options to address presbyopia including various types of corneal intrastromal inlays and laser ablation techniques to generate either a multifocal cornea (“PresbyLASIK”) or monovision. The natural history of presbyopia develops prior to cataracts, and these presbyopic surgeries have been largely studied in phakic patients. Nevertheless, pseudophakic patients may also undergo these presbyopia-compensating procedures for enhanced quality of life. This review examines the published reports that apply these technologies to patients after cataract surgery and discusses unique considerations for this population.

## 1. Introduction

The term “presbyopia” derives from Greek for “old eyes” [1] and refers to the age-related loss of natural accommodation and resulting reduction of baseline near vision around the age of 40 years. As people continue to work and stay active later in life than ever before, their need for quality vision at both near and distance vision is also growing. In fact, the presbyopic population worldwide is predicted to rise to 1.4 billion by 2020 and to 1.8 billion by 2050 [2]. Presbyopia can be compensated by glasses or contact lenses, but there is increasing interest in surgical options. Since presbyopia is caused by progressive elasticity changes in the biological crystalline lens, presbyopic surgeries may either directly replace the lens through an intraocular approach or modify extraocular structures such as the cornea or sclera. This review will focus on corneal-based surgical strategies to treat presbyopia and in particular how these methods have been or may be used in pseudophakic patients. To improve uncorrected near vision in presbyopia, the two major techniques to alter the cornea generally utilize either an intracorneal inlay device or laser refractive surgery.

Corneal inlays are devices that are surgically placed within the corneal stroma of the nondominant eye to change the optical properties of the cornea. Several different types of corneal inlays each take a distinct approach to minimizing presbyopia (Table 1). The best studied inlay to date is the Kamra inlay by Acufocus, which is an opaque polymer ring that employs a pinhole concept to expand the depth of focus [3]. The Raindrop Near Vision Inlay by ReVision Optics is a clear hydrogel implant that increases the anterior corneal curvature to add optical power, with a refractive index approximating that of the cornea [4]. Inlays can also confer differing amounts of refractive power as in the Flexivue Microlens implant by Presbia, which creates a multifocal effect via a central plano zone surrounded by circular rings of plus power [5]. The Icolens by Neoptics AG is another corneal inlay with a bifocal design similar in concept to the Flexivue [6]. Corneal inlays are intended to improve uncorrected near vision but may come at the cost of lowered distance vision or increased glare or haloes [7, 8] or rarely infectious keratitis [9]. However, inlays have been promoted as an additive, removable technology unlike laser refractive surgery which ablates corneal tissue [10]; patients usually return to within +/−1.00 diopter of their preoperative refractive state after corneal inlay removal [3]. It should be noted that intrastromal corneal ring segments (ICRS or Intacs) are another type of corneal implant but are indicated for treatment of keratoconus rather than presbyopia.

Laser refractive surgery on the cornea uses an excimer laser to remodel the corneal curvature in order to improve uncorrected vision and reduce dependency on eyeglasses or contact lenses. One of the most popular techniques, laser-assisted in situ keratomileusis (LASIK), removes corneal stromal tissue under an anterior flap. LASIK can produce either monovision or multifocality, the latter of which has been nicknamed “PresbyLASIK” when it is used to treat presbyopia. Conventional monovision LASIK corrects the dominant eye for distance vision and the nondominant eye for near vision [15]. PresbyLASIK usually follows one of three different approaches to multifocal corneal ablation [16]. In central PresbyLASIK, the central area is shaped hyperpositively for near vision, whereas the midperipheral cornea is adjusted for far vision. In peripheral PresbyLASIK, the central area is shaped for far vision and the midperipheral corneal area for near vision. In the third approach, laser blended vision creates a combination of micromonovision and depth of field increase by inducing spherical aberration. There are concerns for the decreased contrast sensitivity, visual quality, and irreversibility of these laser ablation procedures. Laser blended vision treatment seems to provide the best compromise in terms of safety and quality of vision [16].

## 2. Corneal Inlays for Presbyopia

In contrast to the literature on corneal inlays in phakic patients, there are only a handful of published reports in pseudophakic individuals (Table 2). These include only case reports and retrospective case series. The largest study of pseudophakic patients undergoing inlay implantation included 13 patients with monofocal intraocular lenses (IOLs) [12]. Four of these patients also underwent simultaneous LASIK to optimize their underlying refractive error prior to insertion of a Kamra pinhole inlay. The mean uncorrected near visual acuity (UNVA) improved 5 lines from J10 to J4 at 3 months postoperatively, without significant change in mean uncorrected or corrected distance visual acuity (UDVA, CDVA) or corrected near visual acuity (CNVA). On a postoperative survey, 77% of the patients said they would opt to have the surgery again. In an earlier report by the same group, Kamra inlays were inserted in the nondominant eye of 3 patients with phakic IOL implants [11]. These patients saw 2–5 lines of improvement in UNVA without change in UDVA at 3 months postoperatively. Both of these studies had a short follow-up period of 3 months, at which point the authors assert that operative results would have stabilized. Nonetheless, longer studies are definitely needed as the mean age of these patients was around 55 years and so the patients would be expected to live with the inlays for several decades.

Two case reports with slightly longer follow-up each described a pseudophakic patient who retained improvement of uncorrected near vision at 6–24 months after a corneal inlay procedure. One young patient at 32 years of age had received a monofocal IOL on account of a traumatic cataract in the right eye, and a year later he developed headaches and asthenopia while reading with his right eye [14]. Given his requirement of a +3.00 reading add in the right eye, he could not tolerate glasses due to anisometropia. After Kamra inlay implantation, he reported improved symptoms but also slightly worse reading in dim light compared to bright light conditions. His UNVA improved from J17 to J3 in the operated eye at the 6-month follow-up. Another pseudophakic patient with a monofocal IOL saw a J3 to J1 improvement in UNVA after implantation of a Flexivue Microlens inlay 6 months after the cataract extraction [13]. This recovery of uncorrected near vision continued to be maintained at a 2-year follow-up after inlay implantation. Interestingly, this patient developed a symptomatic posterior capsule opacification in the operated eye at 2 years after the cataract surgery, underwent standard neodymium:YAG (Nd:YAG) capsulotomy, and was reported to still be satisfied and spectacle free with UNVA of J1 6 months later. The authors asserted that the transparent design of this particular inlay enabled visualization for the Nd:YAG capsulotomy and also fundoscopy.

The aforementioned studies demonstrate the efficacy of corneal inlays in presbyopes with a history of prior intraocular and/or refractive surgery. Such a history is an exclusion factor for many other studies on inlays. Similar studies done in phakic patients suggest that the improved uncorrected near vision is achieved for at least 1 to 5 years after corneal inlay implantation. A Mexican prospective study of 30 phakic patients undergoing inlay implantation (Raindrop Near Vision Inlay) and LASIK demonstrated improvement of mean UNVA by 3 lines at one year [4], suggesting that the improved UNVA could also be stable at one year in pseudophakes. A larger retrospective study of 277 eyes in Japan showed similar results at one year after simultaneous Kamra inlay implantation and LASIK, with mean UNVA improving from J8–J10 to J2-J3 depending on patient age [17]. The longest published follow-up of inlays involved an Austrian prospective cohort of 32 emmetropic phakic patients who retained improved uncorrected near vision at 5 years after Kamra inlay implantation [18]. Of note, the mean UNVA enhancement declined somewhat from J1 to J3 between the 3- and 5-year time points; however, CDVA did not change. It is worth mentioning that, by the 5-year follow-up, there were still no biocompatibility concerns and 84% of the patients said they would opt for the surgery again. Several complications occurred, including 2 inlays needed to be recentered, 1 eye that required debridement for epithelial ingrowth, 18 eyes that developed iron deposits by year 3, and 1 inlay that was removed at year 3 due to patient dissatisfaction.

There are several case reports describing the opposite sequence of events, where patients with prior corneal inlay implantation subsequently undergo cataract extraction with intraocular lens placement, either with or without initially removing the inlay. These patients had previously received any one of the three major corneal inlay types, including the Kamra inlay [19], Presbia Microlens inlay [20], and the Raindrop Near Vision Inlay [21]. These anecdotes suggest that cataract surgery can still be successfully performed after inlay implantation, which will be important for those young presbyopes opting for surgical treatment of their presbyopia.

## 3. Laser Refractive Surgery for Presbyopia

An extensive review of the literature on laser refractive surgery for presbyopia revealed very few studies done in pseudophakic patients, although there are numerous published accounts in phakic patients. Similar to the body of literature on corneal inlays, a history of prior intraocular surgery such as cataract extraction typically excluded such patients from studies on laser refractive surgery for presbyopia. Nonetheless, laser refractive surgery has been performed successfully on pseudophakes to correct for ametropias [22–25], suggesting that laser refractive surgery to address presbyopia is likely to be equally successful in pseudophakic patients who have monofocal IOLs. Clearly, the application of laser refractive surgery to presbyopia in pseudophakia merits more research.

Various studies examine the efficacy of laser refractive surgery on treating presbyopia in phakic patients. A prospective trial of central multifocal PresbyLASIK on 50 hyperopic-presbyopic eyes resulted in spectacle independence at all distances for 72% of the patients after 6 months, although nearly a third lost 1-2 lines of CDVA [26]. This and similar studies have suggested that multifocal laser approaches to improving uncorrected near vision in presbyopia may compromise distance vision to some degree [27–29]. Another study utilizing peripheral PresbyLASIK improved mean binocular UDVA and UNVA with high reported patient satisfaction rates but decreased contrast sensitivity [30]. PresbyLASIK treatment for presbyopia may be further modified in pseudophakic patients who are not satisfied with the outcome, as described in a recent case report using a wavefront-guided aspheric treatment to reverse the presbyopic treatment and eliminate dysphotopsia up to 6 months later [29].

LASIK and photorefractive keratectomy (PRK) are known to be effective for fine-tuning residual ametropias after presbyopia-compensating IOL implantation [31], which suggests that laser refractive surgery could similarly address presbyopia after IOL implantation. Sixty-four pseudophakic eyes maintained improved vision at 4 years after LASIK [32]. Two subsequent studies showed efficacy of LASIK at up to 6 months in treating pseudophakic ametropia after multifocal IOL implantation in 53 eyes [33] and 85 eyes with mixed ametropias [23]. PRK has also been used to treat pseudophakic ametropia after multifocal IOL implantation [34]. These and other similar studies suggest that laser refractive surgery could also be used in pseudophakes to treat presbyopia. A general consensus is that LASIK should be performed at least 6–12 weeks after intraocular surgery to reduce possible complications related to cataract incisions, postoperative corneal edema, and refractive and IOL stability [31]. While PRK and LASIK have both been performed safely in pseudophakic patients, it has been postulated that neither are as effective as primary refractive surgery [25]. Conductive keratoplasty (CK), a corneal refractive surgical technique that uses radiofrequency energy to reshape the central cornea by shrinking peripheral collagen, has also been used to treat presbyopia. While again the majority of studies only include phakic patients, one Chinese study reported that CK improved UNVA up to one year later in presbyopic pseudophakic patients with monofocal IOLs [35].

Studies have noted that myopes and hyperopes may express different levels of satisfaction with PresbyLASIK as well as with other presbyopic treatments. Although near acuity results tend to be better in myopes, the majority of hyperopes are satisfied whereas the majority of myopes are not [16]. The decreasing visual quality after PresbyLASIK in myopes who have experienced excellent uncorrected near visual acuity preoperatively can lead to dissatisfaction over unmet expectations.

## 4. Patient Factors and Expectations: Special Considerations for Pseudophakia

Pseudophakic patients present a unique set of challenges and advantages for presbyopic surgeries as compared to the phakic population. Some of the challenges stem from the prior intraocular surgery itself, such as navigating corneal scars and residual corneal irregularities from prior incisions. Additionally, IOL aberrations may alter laser refractive measurements and calculations normally used on phakic eyes [31]. In general, the age difference of approximately two decades between pseudophakic patients and typical refractive patients also renders keratorefractive treatments less predictable and less effective [25]. The older age of most pseudophakic patients correlates with increased prevalence of dry eye symptoms and slower healing rates. Studies have suggested that the final uncorrected visual acuity after excimer laser or corneal inlay surgery is lower in pseudophakic patients than in typical refractive patients [36].

On the other hand, older patients have reported similar or higher levels of satisfaction after presbyopic surgeries as compared with younger patients who may have had higher postoperative acuity expectations [17, 36]. Presbyopia may continue to progress for phakic individuals who undergo refractive or inlay procedures at a younger age, whereas this is not an issue for patients who have had cataract surgery. At the same time, the lack of residual natural accommodative power in pseudophakic patients gives little room for error in presbyopic surgeries. It is worth noting, however, that because the IOL treats most of the spherical error in pseudophakic patients, there would be less keratorefractive-induced effect on the corneal prolate asphericity and quality of vision than in nonametropic phakic patients [25].

In all patients, regardless of phakic status, there are certain considerations to optimize a satisfactory outcome from corneal-based presbyopic surgeries. The importance of a thorough preoperative evaluation cannot be understated, and trial frames or contact lenses should be used in various lighting conditions to help develop realistic patient expectations. Patients should be selected appropriately from baseline characteristics including tear film adequacy, normal corneal shape and thickness, and reasonable expectations. There need to be more studies and counseling of patients on possible negative outcomes of presbyopic surgeries, ranging from increased dry eye or glare symptoms to decreased distance and/or night vision and subsequent safety concerns. Presbyopic surgery may not completely eliminate the need for reading glasses. Given the necessity of fundus viewing for diagnosis and treatment of diseases such as age-related macular degeneration, diabetic retinopathy, or retinal detachments, it should be considered that corneal inlays may obstruct the view or hinder treatment unless they are first removed. Although corneal inlays are promoted as removable, long-term follow-up on removed inlays is not available and PresbyLASIK procedures are in general not reversible with the commonly available technology at present. As the first wave of refractive patients begins to require cataract surgery, we may be required to change not only the assessment and technique of cataract surgery for these patients, but also how we counsel these patients who have high expectations for visual acuity and spectacle independence.

## 5. Conclusions

For patients suffering from presbyopia, there are now exciting surgical alternatives to glasses and contact lenses. Pseudophakic patients who still desire better uncorrected near vision may choose corneal-based surgical therapies, such as corneal inlays or laser refractive surgery. Nevertheless, presbyopic surgeries will not be ideal for all patients, and appropriately screening patients and managing patient expectations are both key to maximizing satisfactory outcomes. Many studies on presbyopic surgeries excluded patients with prior ocular surgery, and the few published studies on pseudophakic patients are limited by short-term follow-up, small numbers, and limited lighting conditions. Undoubtedly, there is a pressing need for more research on presbyopic surgeries in the pseudophakic population.

## Figures and Tables

**Table 1:** Optical principles of corneal inlays.

Optical principle	Corneal inlay
Small aperture	Kamra
Corneal reshaping	Raindrop Near Vision
Refractive optics (multifocality)	Flexivue Microlens, Icolens

**Table 2:** Published studies of corneal inlays in pseudophakic patients.

Publication	Number of patients, IOL type	Technique	Length of follow-up	Change in monocular (and binocular) visual		Illumination conditions
				acuities for uncorrected distance and near vision		
				Change in UNVA (tested at 30 cm)	Change in UDVA (tested at 20 feet unless otherwise indicated)	
Huseynova et al., 2013 [11]	3, phakic IOLs	Kamra (2 patients had prior LASIK)	3 months	2–5 lines (J4 to J2, J6 to J4, and J10 to J5)	No change in mean	Not mentioned

Huseynova et al., 2014 [12]	13, monofocal IOLs	Kamra (4 patients underwent simultaneous or prior LASIK)	3 months	5 lines mean improvement (J10 to J4)	No change in mean	“Standard bright light conditions”

Stojanovic et al., 2014 [13]	1, monofocal IOL	Presbia Microlens	2 years	J3 to J1 (binocular J3 to J1)	20/25 to 20/32 (binocular 20/20, no change)	Not mentioned

Ziaei and Mearza, 2013 [14]	1, monofocal IOL	Kamra (simultaneous LASIK)	6 months	J17 to J3	6/10 to 6/5	Not mentioned

N.B. Intermediate visual acuity was not reported in any of these studies. Except where indicated, binocular visual acuities were not reported in these studies.

## References

[1] Dictionary.com Unabridged presbyopia. http://dictionary.reference.com/browse/presbyopia.

[2] Holden B. A., Fricke T. R., Ho S. M. (2008). Global vision impairment due to uncorrected presbyopia. *Archives of Ophthalmology*.

[3] Yilmaz Ö. F., Alagöz N., Pekel G. (2011). Intracorneal inlay to correct presbyopia: long-term results. *Journal of Cataract & Refractive Surgery*.

[4] Garza E. B., Chayet A. (2015). Safety and efficacy of a hydrogel inlay with laser in situ keratomileusis to improve vision in myopic presbyopic patients: one-year results. *Journal of Cataract & Refractive Surgery*.

[5] Limnopoulou A. N., Bouzoukis D. I., Kymionis G. D. (2013). Visual outcomes and safety of a refractive corneal inlay for presbyopia using femtosecond laser. *Journal of Refractive Surgery*.

[6] Baily C., Kohnen T., O'Keefe M. (2014). Preloaded refractive-addition corneal inlay to compensate for presbyopia implanted using a femtosecond laser: one-year visual outcomes and safety. *Journal of Cataract & Refractive Surgery*.

[7] Tomita M., Kanamori T., Waring G. O. (2012). Simultaneous corneal inlay implantation and laser in situ keratomileusis for presbyopia in patients with hyperopia, myopia, or emmetropia: six-month results. *Journal of Cataract & Refractive Surgery*.

[8] Seyeddain O., Hohensinn M., Riha W. (2012). Small-aperture corneal inlay for the correction of presbyopia: 3-year follow-up. *Journal of Cataract & Refractive Surgery*.

[9] Duignan E. S., Farrell S., Treacy M. P. (2016). Corneal inlay implantation complicated by infectious keratitis. *British Journal of Ophthalmology*.

[10] Lindstrom R. L., MacRae S. M., Pepose J. S., Hoopes P. C. (2013). Corneal inlays for presbyopia correction. *Current Opinion in Ophthalmology*.

[11] Huseynova T., Kanamori T., Waring G. O., Tomita M. (2013). Small-aperture corneal inlay in presbyopic patients with prior phakic intraocular lens implantation surgery: 3-month results. *Clinical Ophthalmology*.

[12] Huseynova T., Kanamori T., Waring G. O., Tomita M. (2014). Outcomes of small aperture corneal inlay implantation in patients with pseudophakia. *Journal of Refractive Surgery*.

[13] Stojanovic N. R., Panagopoulou S. I., Pallikaris I. G. (2014). Refractive corneal inlay for near vision improvement after cataract surgery. *Journal of Cataract & Refractive Surgery*.

[14] Ziaei M., Mearza A. A. (2013). Corneal inlay implantation in a young pseudophakic patient. *Journal of Cataract & Refractive Surgery*.

[15] Braun E. H. P., Lee J., Steinert R. F. (2008). Monovision in LASIK. *Ophthalmology*.

[16] Pallikaris I. G., Panagopoulou S. I. (2015). PresbyLASIK approach for the correction of presbyopia. *Current Opinion in Ophthalmology*.

[17] Tomita M., Waring G. O. (2015). One-year results of simultaneous laser in situ keratomileusis and small-aperture corneal inlay implantation for hyperopic presbyopia: comparison by age. *Journal of Cataract & Refractive Surgery*.

[18] Dexl A. K., Jell G., Strohmaier C. (2015). Long-term outcomes after monocular corneal inlay implantation for the surgical compensation of presbyopia. *Journal of Cataract & Refractive Surgery*.

[19] Tan T.-E., Mehta J. S. (2013). Cataract surgery following KAMRA presbyopic implant. *Clinical Ophthalmology*.

[20] Stojanovic N. R., Panagopoulou S. I., Pallikaris I. G. (2015). Cataract surgery with a refractive corneal inlay in place. *Case Reports in Ophthalmological Medicine*.

[21] Parkhurst G. D., Garza E. B., Medina A. A. (2015). Femtosecond laser-assisted cataract surgery after implantation of a transparent near vision corneal inlay. *Journal of Refractive Surgery*.

[22] Kim P., Briganti E. M., Sutton G. L., Lawless M. A., Rogers C. M., Hodge C. (2005). Laser in situ keratomileusis for refractive error after cataract surgery. *Journal of Cataract & Refractive Surgery*.

[23] Muftuoglu O., Prasher P., Chu C. (2009). Laser in situ keratomileusis for residual refractive errors after apodized diffractive multifocal intraocular lens implantation. *Journal of Cataract & Refractive Surgery*.

[24] Alio J. L., Abdelghany A. A., Fernández-Buenaga R. (2014). Management of residual refractive error after cataract surgery. *Current Opinion in Ophthalmology*.

[25] Sáles C. S., Manche E. E. (2015). Managing residual refractive error after cataract surgery. *Journal of Cataract & Refractive Surgery*.

[26] Alió J. L., Chaubard J. J., Caliz A., Sala E., Patel S. (2006). Correction of presbyopia by technovision central multifocal LASIK (presbyLASIK). *Journal of Refractive Surgery*.

[27] Luger M. H., Ewering T., Arba-Mosquera S. (2013). One-year experience in presbyopia correction with biaspheric multifocal central presbyopia laser in situ keratomileusis. *Cornea*.

[28] Uthoff D., Pölzl M., Hepper D., Holland D. (2012). A new method of cornea modulation with excimer laser for simultaneous correction of presbyopia and ametropia. *Graefe's Archive for Clinical and Experimental Ophthalmology*.

[29] Ang R. E., Reyes R. M. M., Solis M. L. P. (2015). Reversal of a presbyopic LASIK treatment. *Clinical Ophthalmology*.

[30] Pinelli R., Ortiz D., Simonetto A., Bacchi C., Sala E., Alió J. L. (2008). Correction of presbyopia in hyperopia with a center-distance, paracentral-near technique using the Technolas 217z platform. *Journal of Refractive Surgery*.

[31] Macsai M. S., Fontes B. M. (2008). Refractive enhancement following presbyopia-correcting intraocular lens implantation. *Current Opinion in Ophthalmology*.

[32] Zaldivar R., Oscherow S., Piezzi V. (2002). Bioptics in phakic and pseudophakic intraocular lens with the Nidek EC-5000 excimer laser. *Journal of Refractive Surgery*.

[33] Alfonso J. F., Fernández-Vega L., Montés-Micó R., Valcárcel B. (2008). Femtosecond laser for residual refractive error correction after refractive lens exchange with multifocal intraocular lens implantation. *American Journal of Ophthalmology*.

[34] Leccisotti A. (2004). Secondary procedures after presbyopic lens exchange. *Journal of Cataract & Refractive Surgery*.

[35] Ye P., Xu W., Tang X. (2011). Conductive keratoplasty for symptomatic presbyopia following monofocal intraocular lens implantation. *Clinical & Experimental Ophthalmology*.

[36] Kuo I. C., O'Brien T. P., Broman A. T., Ghajarnia M., Jabbur N. S. (2005). Excimer laser surgery for correction of ametropia after cataract surgery. *Journal of Cataract & Refractive Surgery*.

